# The Contribution of Immune and Glial Cell Types in Experimental Autoimmune Encephalomyelitis and Multiple Sclerosis

**DOI:** 10.1155/2014/285245

**Published:** 2014-10-12

**Authors:** Samuel S. Duffy, Justin G. Lees, Gila Moalem-Taylor

**Affiliations:** School of Medical Science, The University of New South Wales, Wallace Wurth Building East, Level 3, Room 327, Sydney, NSW 2052, Australia

## Abstract

Multiple sclerosis (MS) is a chronic inflammatory disease of the central nervous system characterised by widespread areas of focal demyelination. Its aetiology and pathogenesis remain unclear despite substantial insights gained through studies of animal models, most notably experimental autoimmune encephalomyelitis (EAE). MS is widely believed to be immune-mediated and pathologically attributable to myelin-specific autoreactive CD4+ T cells. In recent years, MS research has expanded beyond its focus on CD4+ T cells to recognise the contributions of multiple immune and glial cell types to the development, progression, and amelioration of the disease. This review summarises evidence of T and B lymphocyte, natural killer cell, macrophage/microglial, astrocytic, and oligodendroglial involvement in both EAE and MS and the intercommunication and influence of each cell subset in the inflammatory process. Despite important advances in the understanding of the involvement of these cell types in MS, many questions still remain regarding the various subsets within each cell population and their exact contribution to different stages of the disease.

## 1. Introduction

Multiple sclerosis (MS) is a chronic demyelinating disease of the central nervous system (CNS), which is at present attributable to a self-sustaining autoimmune mechanism. It is the most prevalent disabling neurological disease affecting young people [1] and one of the most common inflammatory conditions of the CNS [2], affecting approximately 2.5 million people worldwide [3]. Whilst the aetiology of MS is largely unknown, genetic, metabolic, environmental, and immunological factors have all been implicated [4]. The main pathological characteristics of MS are CNS plaques composed of inflammatory cells, demyelinated axons, reduced oligodendrocyte numbers, transected axons, and gliosis. Most lesions develop in the white matter but may also be present in areas of grey matter. MS patients show a wide range of neurological symptoms that originate in different areas of the CNS, which may appear as sudden attacks or as a steady progression. Symptoms include motor deficits (e.g., muscular spasms and weakness), sensory disturbances (e.g., paraesthesia) and neuropathic pain, fatigue, visual disturbances, continence problems (e.g., bladder incontinence and constipation), and neuropsychological symptoms (e.g., memory loss and depression) [5]. Although the clinical course of MS is highly variable, several disease subtypes have been described (Table 1) [6–8]. Progressive MS is a highly disabling condition where increasing paralysis renders 50% of patients unable to walk within 25 years of clinical onset [9].

Experimental autoimmune encephalomyelitis (EAE) is a widely accepted animal model of MS that has been used to study the pathophysiology of the disease since first being described in 1933 by Rivers and colleagues [10]. It shares many pathological features with MS, such as chronic neuroinflammation, demyelination, and neuronal damage, and is generated by autoimmune attack on the CNS [11, 12]. Immunisation with self-antigenic epitopes of myelin is used to actively induce an autoimmune response in the CNS of rodents and includes myelin oligodendrocyte glycoprotein (MOG) [13], myelin basic protein (MBP) [14], and proteolipoprotein (PLP) [15], among others. CNS antigens such as these can be highly encephalitogenic and trigger EAE by emulating the characteristic breakdown of the blood brain barrier (BBB) seen in the early stages of MS. This allows multifocal infiltration of activated immune cells into the CNS, which proceed to attack the myelin sheath [16]. An immune response is generally initiated within two weeks of immunisation in the periphery, leading to the typical presentation of ascending paralysis (tail to hind limb to fore limb paralysis) accompanied by a progressive loss in body weight of the animal [17]. EAE represents a range of models with different disease course and pathology, depending on the immunising antigen and the animal species and strain. As such, each EAE model recapitulates a specific repertoire of pathological similarities to those seen in MS. The close clinical and histopathological parallels that can be drawn between specific forms of EAE and MS subtypes suggest EAE to be a useful tool to further our understanding of the mechanisms involved in autoimmunity and may assist in the development of novel therapeutics for MS.

It is worthy to mention, however, that the translational relevance of EAE to MS is highly debated. Despite sharing certain pathogenic features with MS, the unique pattern of demyelination characteristic of MS is not accurately recapitulated in any existing EAE models, and numerous therapies found to be successful in suppressing EAE have often been shown to have limited efficacy in MS. The EAE model also fails in recognising emerging non-autoimmune theories of MS pathogenesis such as virally induced mechanisms and the “inside-out” idea coined by Stys and colleagues [18], whereby MS is proposed to initiate within the CNS as a primary neurodegenerative disorder. The immune response, bringing with it the archetypal inflammatory phenotype seen with MS lesion formation, is suggested to occur secondary to a primary demyelinating event [18, 19]. Theiler's murine encephalomyelitis virus (TMEV) and neurotropic strains of mouse hepatitis virus (MHV) models are the most widely studied representations of virally induced demyelinating disease, whilst models utilising toxins such as cuprizone or lysophosphatidylcholine may be more useful for investigating mechanisms of demyelination and remyelination [20]. Although important to recognise, alternative theories of the aetiology of MS and insights gathered through models other than EAE are beyond the scope of this review. Our focus is to summarise known immune and glial cell-mediated mechanisms of disease pathogenesis, as brought to light through studies utilising various EAE models and in MS patients.

**Figure 1:**
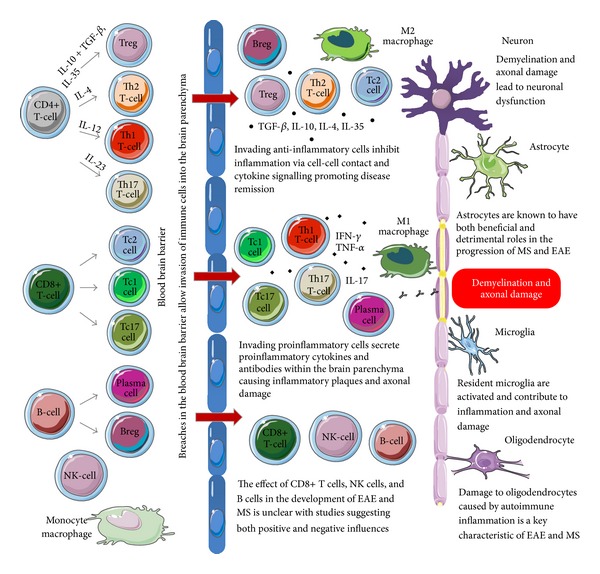
Immune and glial cell subtypes and their contributions to the pathogenesis of EAE and MS. During the development and progression of EAE and MS, a variety of cells representing both the innate and adaptive immune system breach the blood brain barrier and invade the brain parenchyma. Resident glial cells also become activated and play an important role in the pathogenesis of EAE and MS. Some of the cell types involved are proinflammatory and promote demyelination, axonal damage, and the formation of disease plaques, whilst other cell types have anti-inflammatory and/or regulatory properties and inhibit disease progression by facilitating tissue repair.

Currently, MS is most widely thought to be mediated by activation of autoreactive myelin-specific T cells that enter the CNS and initiate a chronic inflammatory response. This is believed to be accompanied by slow neurodegeneration leading to a progressive decrease in neuronal count and grey matter volume over time [21]. Such neurodegeneration becomes increasingly more predominant as the disease enters its later stages and at present is extremely difficult to treat. The hypothesis of MS as a T cell-mediated autoimmune disease is supported by the fact that adoptive transfer of activated myelin-specific CD4+ T cells can induce EAE [22]. As already stated, this view has recently come under criticism; however, it is nonetheless irrefutable that MS possesses a central inflammatory aspect, which will be explored in this review. Specifically, it has become increasingly clear that pathogenesis of MS and EAE expands far beyond the idea of a solely CD4+ T-cell-mediated autoimmune disease. Rather, it involves various immune cells of both arms of the innate and adaptive immune system, as well as immune-like glial cells. In this review, we summarise the current evidence for the involvement of some immune cell subsets and glial cells in EAE and MS (Figure 1).

## 2. Involvement of T Lymphocytes

The lymphocyte population consists of thymus-derived T cells, bone marrow-derived B cells, and natural killer (NK) cells. T lymphocytes are a heterogeneous group of cells that function as part of the adaptive immune system and mediate cellular immunity. They can be divided into three broad categories: T helper (Th/CD4+) cells, cytotoxic (CD8+) cells, and regulatory T (Treg) cells. Within each category, T cells are able to differentiate into distinct subtypes depending on specific cytokine signalling, the expression of chemokine receptors, transcription factors, and epigenetic modifications. Each subset has a different cytokine profile and thus exerts an individualised role in the immune response. T cells are rare within tissue of the intact nervous system but actively infiltrate the CNS of animals with EAE [23, 24] and MS brain and spinal cord lesions [25, 26].

### 2.1. T Helper Cells

CD4+ cells carry out multiple functions including the regulation of innate and adaptive immunity, activation of other immune and non-immune cells, and suppression of immune reactions. MS is commonly conceptualised as being mediated by type 1 T helper (Th1) cells, which differentiate from naïve T cells in response to IL-12 production by antigen presenting cells [27]. Committed Th1 cells produce predominantly proinflammatory cytokines such as interferon- (IFN-) *γ* and tumour necrosis factor- (TNF-) α, which have been implicated in EAE and MS.

Administration of a TNF-receptor-IgG fusion protein, a TNF antagonist, has been shown to prevent clinical signs of actively induced EAE; however, total CD4+ cell infiltration appeared unaltered [28]. The timing of TNF-receptor IgG fusion protein therapy was later shown to be critical, as administration prior to the clinical onset of disease markedly reduced EAE severity and neurological deficit, whilst established clinical disease was relatively refractory to treatment [29]. Additional studies have shown that TNF-deficient C57BL/6 mice induced with MOG_35-55_ develop EAE, albeit with delayed clinical onset and a failure of inflammatory leukocytes to migrate into the CNS parenchyma [30]. Treatment of a relapsing-remitting form of EAE with soluble TNF receptor:Fc/p80 given after disease onset ameliorated both clinical deficit during the initial attack and the exacerbation rate for subsequent attacks [31]. The individual roles of the two TNF receptors have also been investigated, with TNF receptor 1 knockout mice shown to develop less severe EAE characterised by minimal demyelination as compared to WT mice. In contrast, TNF receptor 2 knockout mice developed severe EAE with marked demyelination, pointing to both an inflammatory and anti-inflammatory aspect to TNF action that is dependent on alternative activation of its two receptors [32]. In support of such a notion, soluble TNF receptor 1, a specific inhibitor of TNF-α, is able to suppress the development of EAE passively induced by adoptive transfer of MBP-sensitised T cells [33]. Further, TNF receptor 1-deficient mice show decreased demyelination and protection from clinical disease, suggesting a role for TNF receptor 1 in oligodendrocyte damage [34].

Despite the consensus of the literature suggesting a pathogenic function for TNF in EAE, there is also evidence of a nonessential or even anti-inflammatory role for the cytokine in disease pathogenesis. TNF knockout mice or mice with disruption to the TNF gene have been shown to develop EAE with high mortality and extensive immune cell infiltration and demyelination in the brain and spinal cord [35, 36]. TNF gene inactivation has also been demonstrated to convert otherwise MOG-resistant mice to a state of high susceptibility, and TNF treatment in TNF knockout mice dramatically reduces EAE severity [36]. Other studies have shown TNF deficiency to delay EAE onset, yet the cytokine appears unnecessary for disease progression as severe EAE associated with paralysis, widespread inflammation, and primary demyelination eventually develops to a similar extent to that seen in WT animals [37, 38]. A recent paper testing the effects of TNF-α blockade in MOG_35-55_-induced EAE mirrors the above results by demonstrating that treatment reduced the incidence and delayed the clinical onset of EAE but had no effect on disease severity once established [39]. Attempts at targeting TNF for the treatment of MS have been similarly disappointing. Soluble TNF receptor IgG fusion protein, despite showing success in treating EAE [28, 29], failed to show benefit in alleviating neurological deficit, disease exacerbations, or lesion formation in RRMS patients [40].

The role of IFN-*γ* and IFN-*γ*-producing Th1 cells is also unclear. An early study testing the efficacy of IFN-*γ* as a therapeutic option for MS reported significant disease exacerbation after treatment [41]. Transgenic mice expressing IFN-*γ* in myelinating oligodendrocytes showed no spontaneous CNS inflammation or demyelination and developed EAE in a manner similar to WT mice following disease induction. However, transgenic mice showed chronic neurological deficit as WT mice were experiencing disease remission [42]. Th1 cells expressing IFN-*γ* are known to infiltrate in increased numbers into the brain of mice with EAE [43], and blocking IFN-*γ* production has been shown to inhibit the progression of EAE [44]. On the contrary, there are also reports of mice deficient in either IFN-*γ* or its receptor being susceptible to severe EAE [45, 46], and injection of neutralising antibodies to IFN-*γ* exacerbates both passively and actively induced EAE [47, 48]. IFN-*γ* knockout mice also showed delay in the onset of clinical EAE compared to WT mice; however, the peak of the disease was more severe in the knockout animals, suggesting a protective role for the cytokine in late-stage disease [49]. Autoreactive CD4+ cells collected from RRMS patients exhibit a more differentiated Th1 phenotype compared to healthy controls [50], and relapse is associated with increased production of IFN-*γ* [51]. A double-blind placebo-controlled trial evaluating the efficacy of antibodies to IFN-*γ* and TNF-α in active SPMS found that blockade of IFN-*γ*, but not TNF-α, leads to reduced disability scores, decreased numbers of active lesions, and systemic cytokine changes including increased TGF-*β* production and a decrease in IL-1*β*, TNF-α, and IFN-*γ* concentrations [52]. Findings regarding the role of Th1 cell signature cytokines in EAE and MS are largely discrepant, and further research is needed to ascertain their exact role in the pathogenesis of both diseases.

Type 2 T helper cells (Th2) represent a protective anti-inflammatory subpopulation of T cells which produce cytokines such as interleukins IL-4, IL-5, IL-10, and IL-13 [53]. Th2 cells polarise in response to an environment containing IL-4 [27] and are believed to exert a suppressive role in EAE. Drugs inducing broad upregulation of Th2 cytokines have been shown to ameliorate EAE and result in a parallel blockade of Th1-like responses, including decreases in IFN-*γ*, TNF-α, and IL-12 [54, 55]. The use of IL-4 knockout mice has demonstrated inconclusive findings in determining the exact contribution of the cytokine in EAE. While some studies showed a minimal role for IL-4 in disease progression [56, 57], others reported increased EAE severity accompanied by extensive inflammatory infiltrates in the CNS as well as increased mRNA levels of IFN-*γ*, IL-1, and TNF in IL-4-deficient mice compared to WT littermates [58]. IL-4 gene therapy utilising HSV-1 vectors delivered to the CNS has also shown promise in ameliorating EAE. Improved remission to relapse rates and severity of relapses in relapsing-remitting EAE [59], as well as delayed clinical onset, reduced disease severity, decreased inflammatory infiltrates, and reductions in demyelination and axonal loss in a model of chronic EAE [60] have been demonstrated. More recently, overexpression of GATA3, a transcription factor required for Th2 differentiation, resulted in delayed clinical onset and reduced EAE severity [61]. Studies comparing IL-4 and IL-10 knockout mice suggest a stronger anti-inflammatory contribution of IL-10 in the suppression of EAE. IL-4 deficient mice have been shown to follow a disease course similar to WT littermates, whilst IL-10 deficient mice experience more severe EAE, a lower level of spontaneous recovery, and increases in IFN-*γ* and TNF-α production in response to encephalitogenic peptides [62, 63]. IL-10 gene therapy was also demonstrated to be effective in reversing inflammation-induced paralysis, weight loss, glial activation [64], and susceptibility to EAE induction by active immunisation [65]. Peripheral blood mononuclear cells expressing IL-4 have been shown to be significantly elevated in MS exacerbations and progressive MS over controls [66] but also found to be decreased alongside IFN-*γ* in active and stable MS compared to controls [67]. IL-10 has also been demonstrated to be simultaneously upregulated with IFN-*γ* in peripheral blood mononuclear cells collected from RRMS patients [68]. Interestingly, serum IFN-*γ*, IL-4, and TNF-α, but not IL-10, were found to be elevated during the acute stage of MS as compared to controls [69]. During MS relapse, levels of TNF-α and IL-10 were both upregulated in the CSF and serum [70]. These findings are most likely reflective of the inherent heterogeneity of the immune response in MS and suggest that, rather than a sequential Th1/Th2 paradigmatic pattern of expression, Th1 and Th2 cells are actively involved in the inflammatory milieu at multiple stages of the disease.

In addition to the Th1/Th2 paradigm, the proinflammatory type 17 T helper cell (Th17) population has also been implicated in the pathogenesis of MS, and Th17 cells infiltrate the inflamed CNS of C57BL/6 mice with MOG_35–55_-induced EAE [71, 72]. IL-23, produced predominantly by macrophages and dendritic cells, appears critical for Th17 cell differentiation [73]. There is also evidence that IL-23, and not IL-12 (which promotes Th1 polarisation of naïve T cells), may be the critical regulator of autoimmune inflammation of the brain in EAE [74]. Interestingly, IL-23-modulated CD4+ T cells are able to passively induce EAE and stimulate the production of both IFN-*γ* and IL-17A by myelin-reactive T cells. EAE development in this particular model was determined to be dependent on IFN-*γ*, as IL-17 receptor-deficient hosts exhibited a similar clinical course to WT hosts [75]. Despite this, neither of the signature cytokines produced by Th1 or Th17 cells (IFN-*γ* and IL-17, resp.) appears essential for the development of EAE [76, 77]. Nonetheless, Th17 cells sensitised to myelin antigens such as PLP_139-151_ and MOG_35-55_ are able to induce EAE following adoptive transfer to naive mice, and in many EAE models, this produces a more clinically severe form of the disease than Th1-mediated EAE [78, 79]. Th17 cells produce the proinflammatory cytokine IL-17, and mice lacking IL-17 or its receptor have been reported to show an attenuation of CNS inflammation and a marked suppression of EAE severity [80, 81]. Alternatively, Haak et al. showed that mice lacking IL-17A and IL-17F do not show any major alleviation of clinical disease and conclude that IL-17 has at best a marginal contribution to the progression of EAE [82]. Furthermore, repeated subcutaneous injection of a neutralising antibody for the p40 subunit of IL-12 and IL-23 also fails to protect against the development of new lesions in RRMS patients [83]. On the other hand, increased IL-17 expression has been correlated with active or relapsing MS [84, 85], and IL-17 receptors on BBB endothelial cells in MS lesions have been implicated as a possible mechanism by which immune cells infiltrate the CNS during MS via disruption of BBB tight junctions [86]. Although unclear to what extent, Th17 cells and the cytokine IL-17 appear to be significantly implicated in the pathogenesis of both EAE and MS.

Novel subtypes of T helper cell, such as the IL-9-producing Th9 cell and the IL-22-producing Th22 cell, are also likely to be implicated in the pathogenesis of EAE and MS. At present, their precise role and the extent of their influence in both diseases remain unclear.

### 2.2. Cytotoxic T Cells

Cytotoxic (CD8+) T cells may also contribute to the immune response in EAE, through both the elimination of self-reactive cells or self-antigen sources and the secretion of specific cytokines. Like CD4+ cells, CD8+ cells can be divided into effector subtypes defined by the cytokines they produce, which may be pro- or anti-inflammatory. Tc1 cells, which produce predominantly IFN-*γ*, and Tc17 cells, which produce IL-17, are thought to be proinflammatory in nature and are therefore likely to contribute to the pathogenesis of EAE. Tc2 cells on the other hand appear to have a protective role in autoimmunity due to their ability to produce anti-inflammatory cytokines including IL-4, IL-5, and IL-10 [87].

CD8+ cells are known to be able to migrate to the CNS of mice with EAE; however, their role thereafter is highly debated. Early studies in mice immunised with MBP allude to a protective role of CD8+ T cells in EAE by showing that depletion of CD8+ T cells worsens clinical disease [88, 89]. More recently, passive induction of EAE via adoptive transfer of CD8+ T cells sensitised to MOG has been demonstrated to produce a histologically more severe and progressive form of the disease than active immunisation using MOG antigens [90–92]. CD8+ cells have also been shown to be necessary in disease induction, as CD8+ T cell deficiency in both Lewis rats immunised with MBP [93] and C57BL/6 mice immunised with MOG_35-55_ [94] confers resistance to the development of EAE. Conversely, Bettini et al. (2009) argue that CD8+ T cells have a limited contribution to EAE induction by showing that MOG_35-55_-immunised C57BL/6 mice deficient in CD8+ cells do develop EAE; however, disease severity is significantly decreased when compared to mice retaining CD8+ T cell function [95]. Adding to the confusion, a recent study showed that CD8+ T cells accumulate in the CNS of mice with EAE, but their presence had no effect on the severity of clinical disease, suggesting that it might be an epiphenomenon rather than a disease-relevant feature [96]. As such, the role of CD8+ T cells in EAE is unclear; however, an approach investigating the involvement of specific effector subtypes and their individual roles in CNS autoimmunity would be a valuable addition to the current knowledge.

CD8+ T cells are also prominent cell types in the inflammatory infiltrate in human MS patients and may even outnumber CD4+ T cells in actively demyelinating lesions. Several expanded clones of CD8+ T cell have been found within MS lesions and some of these clones persist for many years in the CSF and blood of the patients [97]. Additionally, CD8+ T cells have been found in increased numbers proximal to demyelinated axons in the CNS, pointing to their active involvement in the inflammatory process [98]. Biopsy samples from early stage MS patients have revealed extensive CD8+ T cell infiltration in the cortex [99], which suggests a role in the initiation of MS. Interestingly, treatment of MS using anti-CD4 did not eliminate IFN-*γ*-producing primed Th1 cells and provided no clinical benefit [100], whilst broader depletion of both CD4+ and CD8+ T cells using anti-CD52 resulted in reduced disability and risk of relapse [101]. The way in which CD8+ T cells act to exacerbate EAE is most likely due to the contribution of proinflammatory subsets such as the Tc1 and Tc17 cell. This is supported by the fact that IFN-*γ* and IL-17-producing CD8+ T cells specific for apoptotic T cell-associated self-epitopes are significantly increased in the CSF of MS patients compared to healthy controls [102]. Further, IL-17A secretion by Tc17 cells has been shown to promote Th17-mediated induction of EAE [103], and Tc17 cells are present within active MS lesions [104]. Taken together, data indicate that CD8+ T cells with strong inflammatory potential are recruited into the CNS during MS, where they contribute to the pathophysiology of the disease.

### 2.3. Regulatory T Cells

Treg cells are regarded as the most potent immunomodulators of the adaptive immune system, where they act to suppress the action of effector T cells and maintain immune homeostasis [105]. Among other markers, Treg cells express CD4, the IL-2 receptor α-chain (CD25), and the forkhead box protein 3 transcription factor (*FoxP3*). Myelin-specific T cells are able to migrate to, and accumulate within, the CNS of animals with EAE [106, 107], and entry into the recovery phase marks a significant increase in* FoxP3*+ cell numbers to levels higher than those in the periphery (e.g., lymph nodes) [108]. Treg cells may inhibit the action of CD4+ cells through cell-to-cell contact-dependent mechanisms resulting in disruption of T cell receptor (TCR-) induced proliferation and reduced transcription of IL-2 [109]. This would have a significant impact on the immune response as IL-2 functions to regulate T cell cycle progression and differentiation into the various effector T cell subtypes [110]. Treg cell-mediated T cell suppression is visible both* in vitro*, where Treg cells have been reported to inhibit the proliferation of a MOG_35–55_-specific T cell line and subsequent IFN-*γ* production, and* in vivo*, where adoptive transfer has been shown to suppress spontaneous EAE induced by MOG_35–55_ [109]. The cytokines IL-10, IL-35, and transforming growth factor- (TGF-) *β* produced by Treg cells have also been cited as possible means by which Treg cells exert their inhibitory function [111, 112]. The anti-inflammatory cytokine IL-35 is perhaps the least explored and has been shown to inhibit effector T cell proliferation and Th17 differentiation. Further, IL-35 is known to suppress a range of autoimmune diseases [113, 114], and adoptive transfer of IL-35-producing Treg cells protects mice from developing EAE [115]. It is interesting to note that several studies have demonstrated that Treg cells are dysfunctional in both EAE and MS, and their impaired immunosuppressive capacity may be key in disease pathogenesis [116–118].

The mechanisms involved in recovery from EAE and remission in MS are somewhat speculative, but a shift from a predominantly proinflammatory cell infiltrate to one characterised by increased migration of immunosuppressive Treg cells is likely to play a major role. Remission in RRMS has been shown to correspond with increased proportions of* FoxP3*+ Treg cells in the blood [119, 120]; however,* FoxP3+* cells are present in very low numbers within MS lesions, and this appears independent of disease activity [121]. In EAE, a proportion of* FoxP3*+ Treg cells are known to arise from neuron-induced conversion from encephalitogenic T cells and are able to effectively control CNS inflammation [122]. Recovery from EAE is associated with an accumulation of antigen-specific* FoxP3*+ Treg cells into the CNS, which are able to suppress the production of IFN-*γ* by MOG-sensitised T cells in coculture [108]. The immunosuppressive action of Treg cell cytokines IL-10 [123] and TGF-*β* [124] on Th1 cell proliferation and function is perhaps the most likely explanation for the concurrent decrease in the presence of CD4+ cells in the CNS. Interestingly, a recently identified* FoxP3* negative subtype of Treg cells characterised by the expression of the transcription factor* FoxA1* has also been shown to reduce the incidence, clinical scores, and severity of CNS inflammation in EAE following adoptive transfer.* FoxA1* expression in CD4+ cells was shown to confer this suppressive function, through caspase-3 associated apoptosis of activated T cells. Further, IFN-*β*, a common treatment for MS, was demonstrated to induce the differentiation and function of the* FoxA1* Treg cell subtype, suggesting a possible* FoxA1*-mediated mechanism for the efficacy of IFN-*β* treatment [125]. Collectively, it can be seen that Treg cells are likely to play a central role in the suppression of both disease initiation and the function of autoreactive T cells; however, a comprehensive characterisation of the mechanism by which this is accomplished is warranted.

## 3. Involvement of B Lymphocytes

B cells function as part of the adaptive immune response, where they predominantly mediate humoral immunity. Mature B cells are characterised by high expression of CD45R and CD19 [126] and have critical roles as both positive and negative regulators of immunity through antibody production, antigen presentation, and the production of cytokines. In EAE, B cells contribute to demyelination through the production of anti-myelin antibodies following differentiation into plasma cells. However, they have also been shown to have a protective function via downregulation of inflammation and the opsonisation of myelin debris which facilitates clearance by phagocytic cells [127, 128]. IgG and IgM have been found in 50–75% of MS patients in acute, chronic active, and chronic inactive lesions and this appears independent of disease duration, clinical disease, or staging. Double immunofluorescence staining showed that IgG and IgM accumulate on axons and oligodendrocytes and are colocalised with complement in demyelinated areas [129]. Lisak et al. demonstrated that B cells isolated from RRMS patients secrete one or more factors toxic to oligodendrocytes, suggesting a pathogenic function in MS [130]. Cytokine production by B cells also accounts in part for their paradoxical role; B cells primed by Th1 cells secrete proinflammatory cytokines including IFN-*γ*, IL-12, and TNFα, whilst B cells primed by Th2 cells secrete cytokines of a more anti-inflammatory nature, such as IL-4 and IL-13 [131, 132].

A regulatory role of B cells in EAE was suggested by studies utilising B-cell deficient (*μ*MT) mice, which failed to spontaneously recover unlike their WT counterparts [133]. Recently, there has been a focus on the use of anti-CD20 to deplete B cells and examine their functional role in EAE. Administration of anti-CD20 prior to EAE induction was shown to induce a substantial exacerbation of disease severity, as well as increased infiltration of encephalitogenic T cells into the CNS [134]. This was attributed to a loss of anti-inflammatory IL-10 production, which was previously shown to be necessary for the regulatory function of B cells in EAE [135]. In addition to IL-10, IL-35 produced by B cells is vital for their protective role; a recent study has shown that mice with B cell-specific loss of IL-35 expression lost their ability to recover from EAE [136]. Another possible mechanism by which B cells regulate EAE independent of IL-10 is via the induction of Treg cell regulation. Regardless of the method used, B cell depletion results in a reduction of peripheral Treg cells [137, 138]. Ray et al. proposed a role for glucocorticoid-induced TNF receptor family-related protein (GITR) as a mechanism by which B cells induce Treg cell proliferation. The study also showed, in contradiction to previous studies citing IL-10 as the primary mechanism of B cell-mediated suppression of EAE, that expression of GITR, but not IL-10, is required for recovery from EAE [139]. Abundant evidence exists for a protective or regulatory role of B cells in EAE; however, further research is needed to ascertain the mechanisms underlying this and whether it can be applied to the treatment of MS.

Conversely, evidence also exists for a pathogenic role of B cells in EAE. Monson et al. used an anti-CD20 antibody to deplete B cells prior to EAE onset and saw a significant suppression of disease onset [140]. Numerous studies utilising anti-CD20 in established EAE have also shown an attenuation of disease severity. This was associated with less severe CNS inflammation and a reduction in MOG-specific Th1 and Th17 cells, suggesting a role for B cells in antigen presentation and CD4+ T cell activation [134, 138]. Further, Barr et al. (2012) have linked the pathogenicity of B cells in EAE with the production of IL-6. B cells from mice with EAE secrete elevated levels of IL-6 in comparison with naive controls, and mice in which B cell-specific IL-6 has been inhibited show less severe clinical disease than mice retaining full B cell function. An IL-6 driven mechanism for B cell pathogenesis may also operate in MS, as B cells isolated from patients with RRMS show elevated levels of IL-6 production compared to those from healthy controls [141]. Taken together, data suggest that B cells, along with their regulatory role in EAE, are vital players in its onset and progression.

## 4. Involvement of Natural Killer Cells

NK cells are major effector cells of innate immunity, where they form the first line of defence against an array of pathogens and tumour cells [142], and regulate the generation of T cell immunity [143]. Immunohistochemical and flow cytometric analyses have revealed that NK cells account for approximately 17% of total infiltrating inflammatory cells in the CNS of mice at the clinical peak of EAE [144], and the majority of evidence suggests a protective role for NK cells in CNS inflammation. IL-2 coupled with an anti-IL-2 monoclonal antibody was shown to dramatically expand NK cells in both the periphery and the CNS, and this leads to an attenuation of CNS inflammation and neurological deficits in SJL mice with EAE [145]. Studies examining the effects of NK cell depletion demonstrate that increased severity of EAE is related to an absence of NK cell-mediated killing of myelin antigen-specific encephalitogenic T cells [146, 147]. Further, mice deficient in CXCR1, a neuronal chemokine receptor involved in NK cell recruitment into the CNS, were found to have increased EAE-related mortality and severity of inflammatory lesions [148]. Interestingly, depletion of NK cells has also been shown to ameliorate clinical EAE; however, this is suggested to result from an absence of NK cell-mediated regulation of T cell immunity leading to a decrease in total lymphocytes reaching the CNS [149]. The mechanism by which NK cell function leads to a diminution of EAE is largely unexplored but has recently been linked to IgG-induced induction of Treg cells and subsequent suppression of IFN-*γ* and IL-17 production by autoreactive T cells [150]. The production of protective neurotrophic factors by NK cells (along with T cells) has also been cited and appears to support recovery of lesioned spinal motoneurons in EAE [151]. Taken together, it is clear that NK cells are an important regulator of immunity during EAE.

The role of NK cells in MS is also unclear; however, evidence suggests that they may be somewhat dysfunctional in such a context. Hamann et al. showed that NK cell frequency in the CSF during MS is significantly decreased compared to the blood, and that these central NK cells display an immature phenotype [152]. NK cell functional activity is also significantly lower in RRMS patients, which is especially apparent immediately preceding the development of both new and enlarging active lesions [153]. RRMS patients also display significantly diminished cytokine-driven accumulation of IFN-*γ*-producing CD56_bright_ NK cells in the blood, a marker for NK cells possessing increased regulatory function [154]. Interestingly, PPMS and SPMS patients show increased percentages of CD56_dim⁡_ NK cells in the blood, which points to an upregulation of NK cells possessing increased cytotoxic, rather than immunoregulatory function [155]. Further, IFN-*β*, a standard treatment for RRMS, has been shown to expand CD56_bright_ immunoregulatory NK cells [156] and increases the proportion of NK cells in the active phase of the cell cycle [157]. Whether NK cells are directly involved in the therapeutic effect of IFN-*β* treatment is not known. Treatment with daclizumab, a humanized neutralizing anti-CD25 antibody, also selectively expands and activates CD56_bright_ cells and correlates with an inhibition of MS brain lesion activity and a contraction of absolute T cell numbers [158, 159]. Similarly, patients with SPMS that show clinical response to mitoxantrone treatment are associated with not only persistent NK cell enrichment but also increased NK cell maturation [160]. The fact that successful immunomodulatory therapies appear to correlate with a rescue of NK cell function in MS patients suggests a regulatory role for NK cells in MS; however, the exact mechanisms by which this is accomplished remain undefined.

## 5. Involvement of Macrophages and Immune-Like Glial Cells

### 5.1. Macrophages/Microglia

Despite being commonly conceptualised as a T cell-mediated disease, the CNS of both MS patients and EAE models is also characterised by activation of resident microglia, as well as extensive infiltration of monocyte-derived macrophages [161–163]. Recent studies have demonstrated that monocyte-derived macrophages and microglia are functionally distinct populations [164] with unique origins; macrophages develop from self-renewing hematopoietic stem cells in the bone marrow via blood monocyte intermediates, whereas microglia are derived from hematopoietic cells in the yolk sac that migrate into the CNS prior to formation of the BBB [165]. During EAE, activated macrophages within CNS lesion sites were historically difficult to distinguish from activated microglia as both appear similar histologically and share similar antigenic markers, notably ionized calcium-binding adapter protein (IBA-1), major histocompatibility complex class II (MHC II), CXCR1, and CD11b [163, 166, 167]. Early studies used CD45 to distinguish between macrophages and microglia, a method where resident microglia are separated from CD45^hi^ macrophages in haematogenous preparations based on comparatively low expression of CD45 [168, 169]. Recently, however, it has become possible to distinguish between resident microglia and blood-derived macrophages using chimeric mice, whereby bone marrow (BM) cells of naive mice are replaced by donor BM cells containing mismatched-MHC or fluorescently labelled myeloid cells [170, 171]. Using this method, it has been shown that during EAE, a subpopulation of microglial cells became activated in the CNS in the early stages of disease, before clinical symptoms and before the infiltration of peripheral monocytes/macrophages into the CNS [163]. Although suggestive of an active role for microglia in the pathogenesis of EAE, to the best of our knowledge, the differential functional role of microglia and blood-derived macrophages in EAE and MS has not been elucidated to date.

The consensus is that macrophages/microglia play a pathogenic role in both EAE and MS. Bhasin et al. revealed that the timing of macrophage/microglial activation is critical for the progression of EAE. Macrophage inhibitory factor was used to inhibit macrophage/microglial activation and showed that intervention prior to disease induction had only modest effects on EAE progression, whilst intervention at EAE onset significantly ameliorated disease symptoms [172]. Strong correlations have also been found between macrophage infiltration and progression to paralytic EAE, further reinforcing a role for macrophages/microglia in late-stage disease [162]. Similar studies involving inactivation of macrophages/microglia in established EAE have been achieved through targeting estrogen receptor *β* [173] and through the use of microRNA-124 [174], both resulting in ameliorated disease severity and enhanced recovery.

Further subtypes of macrophage/microglia exist, including the predominantly proinflammatory M1 cell (iNOS+) which secretes cytokines including TNF-α and IL-1*β* and the M2 cell (Arg1+), which is anti-inflammatory in nature and is associated with the secretion of IL-10 [175–177]. Unique stimuli endow macrophages/microglia with their phenotype and effector function. Ding et al. first showed that lipopolysaccharides and the proinflammatory cytokine IFN-*γ* promote differentiation of M1 cells [178], whilst differentiation into the M2 subtype is promoted in an anti-inflammatory environment containing IL-4 and IL-13 [179]. M1 and M2 cells have been shown to predominate differentially during the course of EAE, with M1 cells contributing to the establishment of early inflammation in EAE [180]. Indeed, the presence of M1 cells in inflammatory lesions appears to correlate with increased EAE severity, whilst increased M2 cell levels are associated with ameliorated clinical disease and a resolution of inflammation [180–182]. Recent studies have shown that suppression of CNS accumulation of M1 macrophages through conditional ablation of astroglial CCL2 reduces disease severity and preserves axons in EAE [183]. Additionally, adoptive transfer of IL-4-activated M2 cells mitigates clinical disease and inhibits CD4+ T cell activation in mice with EAE [184]. Further research is needed to accurately ascertain the influence of macrophage/microglial subtypes in neuroinflammation associated with EAE and whether their action may provide a basis for the development of novel immunotherapeutics in MS.

### 5.2. Astrocytes

Astrocytes perform an array of homeostatic functions within the CNS, including maintenance of the BBB and modulation of neuronal connections, and are believed to be involved in intercellular communication. Astrocytes have also been implicated in the development of EAE; however, their exact role in CNS inflammation is somewhat unclear due to conflicting studies. Astrocytes are known to be critical in the orchestration of leukocyte recruitment during autoimmune-induced CNS inflammation [185] and are thus vital to the development of EAE. Accumulations of hypertrophic, highly glial fibrillary acidic protein (GFAP) positive astroglia are prominent in the spinal cord during EAE [186], yet whether they serve to positively or negatively modulate disease progression is unknown. Evidence for an aggravatory role has been well demonstrated by studies testing inactivation of the key astroglial transcription factor nuclear factor-kappa B (NF-*κ*B). Inhibition of astroglial NF-*κ*B leads to improved functional outcomes in EAE [187], as well as reduced infiltration of proinflammatory T cells in acute EAE, reduced numbers of macrophages/microglia in chronic EAE, and increased remyelination [188]. The timing of the astrocytic contribution to EAE pathophysiology is not well established; however, astrocytic responses have been shown to coincide with early inflammation and axonal injury [189].

Paradoxically, evidence also exists to suggest a protective role for astrocytes in EAE and MS. Astrocytes expressing the radial-glia cell marker brain lipid binding protein are evident in high numbers within early MS lesions but are significantly less present in chronic lesions seen in long-term sufferers [190]. Wang et al. used transgenic C57BL/6 mice selectively lacking the astrocytic Fas ligand to demonstrate the importance of astrocytes in the control of autoreactive T cells in the CNS of mice with EAE. These mice failed to induce apoptosis of Fas+ CD4+ T cells and did not show an increase in Treg cell numbers beyond the clinical peak of the control group [191], an otherwise common observation in the recovery phase of EAE [108]. Loss of astrocytic leptin signalling has also been shown to have aggravating effects on EAE, with leptin receptor knockout mice recording significantly higher clinical scores than control animals, which was accompanied by increased CD4+ cell infiltration and demyelination [192]. Further, inhibiting the activity of astrocytes in established EAE has been shown to affect the nature of immune cell infiltration and subsequent disease severity. Inhibition of reactive astrocytosis produced increased clinical scores, as well as a substantial increase in myeloid cell infiltration (predominantly macrophages); however, there was no significant change in T cell infiltration when compared to control mice [193]. Astrocytes are also believed to play a role in neuroprotection, and inhibition of IFN-*γ* signalling to astrocytes increases demyelination at the acute peak of EAE, which is followed by diminished clinical remission, increased mortality, and sustained astrocyte activation within the grey matter at later stages of the disease [194]. Taken together, it can be seen that astrocytes play a role in the control of multiple cell types involved in the production of EAE and are therefore likely to be implicated at multiple stages of both EAE and MS.

## 6. Oligodendrocytes

Oligodendrocytes are specialised cells of the CNS that wrap axons with myelin and allow for the efficient conduction of nervous impulses. Oligodendrocyte damage and apoptosis in response to CNS autoimmune inflammation are most widely considered the pathological basis of EAE and MS and are believed to result from the elaboration of proinflammatory mediators and nitric oxide (NO) by activated T cells, macrophages, and activated glial cells [195]. There is abundant evidence suggesting that NO levels are significantly raised within MS lesions [196, 197], and this has been demonstrated to form an important facet of MS-associated oligodendrocyte damage [198]. Despite this, the molecular mechanisms underlying oligodendrocyte dysfunction and death in MS are poorly understood.

Although traditionally viewed as immune targets secondary to a dysregulated immune reaction, emerging evidence also suggests that oligodendrocytes may actively participate in the neuroimmune network. Protection from the adverse effects of the Th1 cytokine IFN-*γ* has been cited as the mechanism by which pancreatic endoplasmic reticulum kinase (PERK) activation within (re)myelinating oligodendrocytes enhances their survival in EAE [199, 200]. The transcription factor interferon regulatory factor 1 (IRF-1) has also been implicated in the pathogenesis of EAE and MS, and transgenic mice with suppressed IRF-1 specifically in oligodendrocytes are protected against EAE and show decreased inflammatory demyelination, as well as oligodendrocyte and axonal preservation. This conveyed protection was related to impaired expression of immune and proapoptotic genes, suggesting that IRF-1 mediates the oligodendrocyte response to CNS inflammation and resulting injury [201].

Remyelination originates from oligodendrocyte precursor cells (OPCs), which may form a reservoir for the differentiation and migration of mature oligodendrocytes into the CNS during demyelinating diseases, such as MS. Spontaneous remyelination is evident in some MS patients, with 60–96% of global lesion areas remyelinated in 20% of MS sufferers at autopsy, and is evident in both RRMS and progressive MS cases [202]. The adult mammalian CNS contains glial precursor cells that express the NG2 proteoglycan, which are known to descend from OPCs in the perinatal CNS. NG2+ cells generate myelinating oligodendrocytes and a limited number of astrocytes to the postnatal brain in EAE [203]. Interestingly, NG2+ cells and mature oligodendrocytes are decreased within areas of subpial cortical demyelination in chronic but not early stage MS [204]. Höftberger et al. have previously proposed that oligodendrocyte loss in later stage MS results from impaired differentiation, migration, and activation capacity of precursor cells [205]. Replenishing the oligodendrocyte precursor pool through intraventricular injection of OPCs derived from human embryonic stem cells [206], or of neural stem cells primed to differentiate into OPCs [207], has been shown to abrogate clinical EAE in mice. Further, Kim and colleagues found that following injection of OPCs, there was subsequent generation of CD45+ cells (a marker for microglia/macrophages), an accumulation of inflammatory cells in the subarachnoid space, and increased numbers of Treg cells in the spinal cord and spleen, suggesting an influence of OPCs on additional immune cell types which may have contributed to the observed decrease in EAE severity [206].

The mechanisms underlying the failure of OPCs to remyelinate damaged axons in MS are relatively unknown. NG2+ cells appear to compensate for demyelination in early EAE (20 days after induction); however, numbers of NG2+ cells and mature oligodendrocytes are strongly diminished in the cerebral cortex in late-stage disease (39 days after induction) [208]. Animal models of EAE suggest that OPCs rather than mature oligodendrocytes are responsible for remyelination in MS, and OPCs have been noted to be significantly more susceptible to injury than mature oligodendrocytes in the context of actively demyelinating MS lesions and* in vitro *stress conditions [209]. Kang et al. have previously reported that inhibition of IL-17 receptor signalling in neuroectodermal CNS resident cells (neurons, astrocytes, and oligodendrocytes) attenuates the severity of EAE in mice [185]. IL-17-mediated disease exacerbation was recently linked to NG2+ cells, which have been cited as the major CNS cellular target of IL-17 in EAE, suggesting a direct relationship between inflammation and neurodegeneration in MS [210]. Both EAE and MS are associated with an inhibition of OPC differentiation into mature oligodendrocytes capable of remyelination and disease remission, and approaches targeting the rectification of this defect may offer a regenerative approach to the treatment of MS.

## 7. Conclusions

It is well established that EAE is induced via autoimmune attack on myelin, leading to inflammatory demyelination and further neurodegeneration. Central aspects concerning the pathogenesis of MS have become highly debated in recent times, especially concerning its underlying aetiology and initiating events. There is nevertheless a broad selection of parallels that may be drawn concerning immune and glial contributions to the pathophysiology of both conditions. Whether an autoimmune mechanism underlies MS is unknown; however, demyelination has an undeniable link to inflammation. This is a pathogenically complex process resulting from the interaction of multiple cell types with encephalitogenic and/or regulatory potential and the immunomodulatory factors they produce (Figure 1). Emerging discoveries concerning different subsets within each cell population and the differential role they may play in the various stages of disease make it difficult to assess their exact role in EAE and MS, with some studies producing conflicting reports. As such, future research is needed to address the specific role of different immune and glial cell subsets in the local inflammatory microenvironment within the CNS longitudinally, which may result in novel therapeutic strategies for MS.

## Figures and Tables

**Table 1:** Subtypes of multiple sclerosis.

Disease subtype	Characteristics	Disease activity
Clinically isolated syndrome (CIS)	First clinical presentation of a disease that shows characteristics of inflammatory demyelination that could be MS but has yet to fulfil criteria of dissemination in time.	CIS and RRMS may be (i) not active, (ii) active (determined by clinical relapses and/or magnetic resonance imaging MRI activity). Active CIS may become RRMS upon fulfilling MS diagnostic criteria.
Relapsing remitting MS (RRMS)	Clearly defined disease relapses with full recovery or with residual deficit upon recovery. Accounts for approximately 80–85% of MS patients.	

Primary progressive MS (PPMS)	Progressive accumulation of disability from onset. Accounts for 10–15% of MS patients.	(i) Active and with progression (measured by clinical evaluation) (ii) Active but without progression (iii) Not active but with progression (iv) Not active and without progression (stable disease).
Secondary progressive MS (SPMS)	Progressive accumulation of disability after an initial relapsing disease course. Afflicts up to 90% of RRMS sufferers after 25 years.	
